# Neuropsychological function at first episode in treatment-resistant psychosis: findings from the ÆSOP-10 study

**DOI:** 10.1017/S0033291718002957

**Published:** 2018-10-23

**Authors:** Eugenia Kravariti, Arsime Demjaha, Jolanta Zanelli, Fowzia Ibrahim, Catherine Wise, James H. MacCabe, Abraham Reichenberg, Izabela Pilecka, Kevin Morgan, Paul Fearon, Craig Morgan, Gillian A. Doody, Kim Donoghue, Peter B. Jones, Anil Şafak Kaçar, Paola Dazzan, Julia Lappin, Robin M. Murray

**Affiliations:** 1 Psychosis Studies Department, Institute of Psychiatry, Psychology & Neuroscience, King's College London, 16 De Crespigny Park, London SE5 8AF, England, UK; 2 Academic Rheumatology Department, School of Immunology & Microbial Sciences, King's College London, Weston Education Centre, 10 Cutcombe Road, London SE5 9RJ, England, UK; 3 Environmental Medicine and Public Health Department, Icahn School of Medicine at Mount Sinai, 1 Gustave L. Levy Place, New York NY 10029-5674, USA; 4 Department of Psychology, University of Westminster, 115 New Cavendish Street, London W1W 2UW, England, UK; 5 Department of Psychiatry, St. Patricks University Hospital and Trinity College, University of Dublin, James St., Dublin 8, Ireland; 6 Health Service and Population Research Department, Institute of Psychiatry, Psychology & Neuroscience, King's College London, 16 De Crespigny Park, London SE5 8AF, England, UK; 7 Division of Psychiatry and Applied Psychology, Queen's Medical Centre, University of Nottingham, Nottingham NG7 2UH, England, UK; 8 Addictions Department, Institute of Psychiatry, Psychology & Neuroscience, King's College London, 16 De Crespigny Park, London SE5 8AF, England, UK; 9 Department of Psychiatry, University of Cambridge, Herchel Smith Building, Cambridge CB2 0SZ, England, UK; 10 Koç University, School of Medicine, Rumelifeneri Yolu 34450 Sarıyer, Istanbul, Turkey; 11 UNSW Research Unit for Schizophrenia, School of Psychiatry, The University of New South Wales, Sydney NSW 2052, Australia

**Keywords:** Cohort study, first episode, neuropsychological, population-based, psychosis, schizophrenia, treatment resistant

## Abstract

**Background:**

Neuropsychological investigations can help untangle the aetiological and phenomenological heterogeneity of schizophrenia but have scarcely been employed in the context of treatment-resistant (TR) schizophrenia. No population-based study has examined neuropsychological function in the first-episode of TR psychosis.

**Methods:**

We report baseline neuropsychological findings from a longitudinal, population-based study of first-episode psychosis, which followed up cases from index admission to 10 years. At the 10-year follow up patients were classified as treatment responsive or TR after reconstructing their entire case histories. Of 145 cases with neuropsychological data at baseline, 113 were classified as treatment responsive, and 32 as TR at the 10-year follow-up.

**Results:**

Compared with 257 community controls, both case groups showed baseline deficits in three composite neuropsychological scores, derived from principal component analysis: verbal intelligence and fluency, visuospatial ability and executive function, and verbal memory and learning (*p* values⩽0.001). Compared with treatment responders, TR cases showed deficits in verbal intelligence and fluency, both in the extended psychosis sample (*t* = −2.32; *p* = 0.022) and in the schizophrenia diagnostic subgroup (*t* = −2.49; *p* = 0.017). Similar relative deficits in the TR cases emerged in sub-/sensitivity analyses excluding patients with delayed-onset treatment resistance (*p* values<0.01–0.001) and those born outside the UK (*p* values<0.05).

**Conclusions:**

Verbal intelligence and fluency are impaired in patients with TR psychosis compared with those who respond to treatment. This differential is already detectable – at a group level – at the first illness episode, supporting the conceptualisation of TR psychosis as a severe, pathogenically distinct variant, embedded in aberrant neurodevelopmental processes.

## Introduction

Schizophrenia and other psychoses are severe neuropsychiatric disorders, heterogeneous in aetiology, clinical trajectory and treatment response (Fanous and Kendler, 2005; van Os, 2016). In approximately 30% of schizophrenia patients, psychotic symptoms respond poorly, if at all, to most antipsychotics, with the notable exception of clozapine (Elkis and Buckley, 2016; Gillespie *et al*., 2017; Howes *et al*., 2017). This emerged as the gold-standard pharmacological intervention for ‘treatment-resistant schizophrenia’ (TRS) in 1988 (Kane *et al*., 1988). Since then, studies have varied considerably in their definitions of TRS (Howes *et al*., 2017), although there is a consistent minimum requirement of two periods of adherence to two different antipsychotics, each administered at adequate doses (variously defined) for at least 4 weeks, resulting in symptom reductions of <20% (Gillespie *et al*., 2017).

Recent evidence suggests that TRS is neurobiologically and categorically distinct from treatment-responsive schizophrenia (Gillespie *et al*., 2017). Unlike treatment-responsive patients, treatment-resistant (TR) ones do not exhibit an elevation in dopamine synthesis capacity (Demjaha *et al*., 2012), and instead, show elevated glutamate levels in the anterior cingulate cortex (Demjaha *et al*., 2014). In addition, previous findings from the ÆSOP-10 study (Aetiology and Ethnicity in Schizophrenia and Other Psychoses) by our group showed that 84% of TR patients were resistant from illness onset (primary TRS), while the remaining 16% made an initial response to antipsychotics, but became TR later (secondary TRS) (Demjaha *et al*., 2017). Lally *et al*. (2016) showed similar results in the GAP (Genetics and Psychosis) first-episode study, with 70% of TR patients having primary TRS.

Despite emerging evidence that specific combinations of cognitive deficits define disease heterogeneity as related to treatment response (Gilbert *et al*., 2014), neuropsychological investigations largely remain an untapped resource for characterising the origin and mechanism of TRS. To date, only six studies have compared neuropsychological function between TR and treatment-responsive patients with psychosis, showing the former to have consistent relative deficits in verbal domains, such as language, verbal intelligence, verbal memory, verbal fluency and verbal interference (Joober *et al*., 2002; de Bartolomeis *et al*., 2013; Iasevoli *et al*., 2016), less consistently reported deficits in nonverbal domains, such as performance intelligence, processing speed, visuospatial function and visual memory (Joober *et al*., 2002; Bourque *et al*., 2013; Frydecka *et al*., 2016), and no cognitive differences from treatment-responsive patients in one study (Anderson *et al*., 2015).

All the above studies have involved cross-sectional investigations of chronically ill samples with schizophrenia or schizoaffective disorder, and with established group differences in treatment response and medication profiles. The respective research designs and methodologies have allowed limited conclusions with regard to two important questions: (1) Do neuropsychological differences between TR and treatment-responsive individuals reflect premorbid differences or the impact of non-remitting psychosis? (2) Do findings from TRS generalise to other psychoses?

To address these questions, we examined baseline neuropsychological data from ÆSOP-10, a population-based study of first-episode psychosis (FEP) with a 10-year follow-up (Fearon *et al*., 2006; Morgan *et al*., 2006, 2014). All neuropsychological assessments were carried out during the patients’ first episode of psychosis, approximately 10 years before participants were characterised as TR or treatment responsive following detailed re-constructions of their case histories by the ÆSOP team (Demjaha *et al*., 2017). In line with the TRS literature and with additional neuropsychological findings in support of dimensional models of psychosis (Kravariti *et al*., 2012), we predicted that TR patients would show deficits in verbal tasks of intelligence, fluency and memory compared with treatment-responsive patients and community controls, both among participants with schizophrenia and in the extended sample with various psychoses.

## Methods

### The ÆSOP-10 study

The present analysis included baseline neuropsychological, sociodemographic and clinical data from ÆSOP-10 (Aetiology and Ethnicity in Schizophrenia and Other Psychoses), a 10-year longitudinal follow-up, population-based study of FEP (Fearon *et al*., 2006; Morgan *et al*., 2006). The study identified all individuals aged 16–65 years with FEP [codes F20–F29 and F30–F33 in the International Classification of Diseases, 10th Revision (ICD-10) manual (World Health Organisation: WHO, 1992*a*)], who presented to specialist mental health services in tightly defined catchment areas in Southeast London, Nottingham and Bristol between September 1997 and August 2000. Exclusion criteria were the previous contact with health services for psychosis, organic causes of psychotic symptoms, transient psychosis due to acute intoxication (as defined by ICD–10) and IQ<50. [Due to the primary focus of this analysis on neuropsychological functions, we used a higher threshold of inclusion herein: IQ>69, as assessed by the Wechsler Intelligence Scale-Revised (WAIS-R; Wechsler, 1981)]. The study further included a random sample of community controls with no past or present psychotic disorder, recruited using mainly a sampling method that matched cases and controls by area of residence. Across the three centres, 568 cases with consensus diagnoses of psychotic illness who met the study inclusion criteria, and 412 community controls, were identified. Patients provided detailed contact information for themselves, their General Practitioners (GPs) and relatives, and consent to be re-contacted for follow-up. Ethical approvals for the baseline and follow-up studies were obtained from local research ethics committees. Detailed overviews of the ÆSOP study design and procedures have been published elsewhere (Fearon *et al*., 2006; Morgan *et al*., 2006, 2014; Demjaha *et al*., 2017).

### Baseline assessment of sociodemographic and clinical characteristics

Sociodemographic data were collected by interviews with the participants using the Medical Research Council Sociodemographic Schedule (Mallett, 1997). Information gaps were filled using additional data sources, including case notes and other informants. Clinical data were collected as soon as possible after the first contact with psychiatric services using the Schedules for Clinical Assessment in Neuropsychiatry (SCAN; WHO, 1992*b*). The SCAN incorporates the Present State Examination (PSE) – Version 10, which was used to elicit symptom-related data at presentation. Where a patient interview was not possible, case notes and, when available, information from informants, were used to complete the SCAN Item Group Checklist. Baseline symptom scores were further subjected to factor analysis, giving rise to five psychopathological dimensions: manic, reality distortion, negative, depressive and disorganization symptom dimensions (for full details, see Demjaha *et al*., 2009). Patients’ ICD–10 diagnoses were determined using the SCAN data on the basis of consensus meetings involving a principal investigator (PI) and other members of the research team with satisfactory interrater reliability (*κ* values ranged from 0.63 to 0.75, *p* < 0.001). Duration of untreated psychosis (DUP) was defined as the period from the onset of psychosis to the first contact with statutory mental health services (for full details, see Morgan *et al*., 2006). Data on illicit substance use before the first presentation to mental health services were collected retrospectively using an ad hoc secondary data collection schedule, which collated data on prevalence and type of illicit substance use from relatives or carers, from the SCAN (WHO, 1992*b*) and from clinical case notes. Controls were screened for psychosis using the Psychosis Screening Questionnaire (Bebbington and Nayani, 1995). Those with a positive rating were further assessed using the SCAN (WHO, 1992*b*) and, where appropriate, excluded.

### Baseline neuropsychological assessment

The present analysis included neuropsychological data collected at baseline using the National Adult Reading Test-Revised (NART-R) (Nelson and Willison, 1991), assessing premorbid intelligence; the Vocabulary, Comprehension, Block Design, and Digit Symbol subtests of the WAIS-R (Wechsler, 1981), assessing verbal and non-verbal intelligence; trials 1–5 and 7 of the Rey Auditory Verbal Learning Test (RAVLT) (Spreen and Strauss, 1991), assessing immediate and delayed verbal recall and learning; the immediate Visual Reproduction trials of the Wechsler Memory Scale – Revised (WMS-R) (Wechsler, 1987), assessing visual memory; the Trail Making A (Reitan, 1958), Trail Making B (Reitan, 1958) and Letter-Number Span (Gold *et al*., 1997) tests of processing speed, working memory and executive function; and Category and Letter Verbal Fluency (category: ‘body parts’, ‘fruits’ and ‘animals’; letter: F, A, S), assessing verbal ability and executive control.

### Follow-up clinical assessment

At approximately 10 years after inclusion, we made an attempt to trace, re-contact and re-assess 557 ÆSOP participants with psychosis, who had initially been identified in the Southeast London and Nottingham centres. Using an extended version of the WHO Life Chart Schedule (LCS) (WHO, 1992*c*) and a Medication History Timeline, comprehensive information on psychopathology, all prescribed antipsychotic medications (start and end date, dosage, adherence, reasons for change or termination), substance use and contact with mental health services was collected and rated for the entire follow-up period using a wide range of information sources: medical case records, follow-up interview with participant or informants (where possible), treating clinicians, ward and community prescriptions, medication charts and clinical documentation (including, where available, reports of drug level testing, and correspondence from the prescribing clinician/to GPs). Using the above sources, adherence to each prescribed antipsychotic throughout the period it was prescribed was rated on a three-point scale (1: 0–33%; 2: 34–67%; 3: 68–100%), using 68% as the cut-off for ‘adherence’. Presence of symptoms at follow-up was further assessed using the Scan – Version 2 (WHO, 1994). Based on all available information, case histories were reconstructed for the entire follow-up period to complete all sections of the Life Chart. A detailed overview of the follow-up clinical assessment procedures has been published elsewhere (Morgan *et al*., 2014; Demjaha *et al*., 2017).

### Representativeness of the follow-up sample

Of the 557 cases who were initially recruited, 434 (78%) underwent follow-up assessments. There were no marked differences in gender, ethnicity, duration of untreated psychosis, or diagnosis between the cases who underwent follow-up assessments and those who were lost to follow-up, except that the follow-up sample was younger (*t* = 2.5; *p* = 0.02).

### Criteria for treatment response and treatment resistance

‘Response to treatment’ was defined as a state of no or mild symptoms (SCAN score<2), not interfering with daily functioning, lasting at least 6 months (Andreasen *et al*., 2005). In line with National Institute for Health and Care Excellence (NICE) criteria (National Institute for Health and Care Excellence, 2014), patients were classified as ‘Treatment Resistant’, if, despite recorded adherence to medication, they continued to show positive symptoms of at least moderate severity (SCAN score⩾2) following two sequential trials of antipsychotic medication at a daily dose of 400–600 mg of chlorpromazine equivalence, each lasting at least 4 weeks. Patients were classified as ‘Treatment Resistant from Illness Onset’ (TRO) if they met resistance criteria after the first two trials of antipsychotic medication, and as ‘Delayed Onset Treatment Resistant’ (DOTR), if such criteria were met after a period of response to treatment. An individual meeting treatment resistance criteria, but later meeting treatment response criteria, would have been classified as treatment resistant. However, the ÆSOP-10 Study did not identify individuals whose response to medication improved during the course of illness. Of the 50 patients who received clozapine (by definition TR), 14 (28%) were clozapine responders, 12 (24%) were clozapine resistant, and the remaining 24 (48%) could not be classified, due to a suboptimal clozapine trial or insufficient clinical and response data (Demjaha *et al*., 2017).

### Representativeness of the follow-up sample that was evaluated for treatment response

Of the 434 cases who were assessed at follow-up, 212 (49%) met criteria for treatment response, 74 (17%) met criteria for treatment resistance (of whom 62: 84% were TRO) and 37 (9%) had never received an adequate trial of antipsychotic medication and could not be included in either category. The remaining 111 participants (26%) had incomplete clinical information documentation and could not be classified. Cases with complete information did not differ notably from the remainder of the follow-up sample in terms of age, gender, ethnicity, DUP or diagnosis (Demjaha *et al*., 2017).

### Statistical analysis

#### Data reduction and generation of composite neuropsychological scores

To avoid the caveats of multiple testing and of experimenter assumptions during the grouping of cognitive tasks into overarching constructs (e.g. executive function), we reduced an original set of 13 neuropsychological variables to a small number of components, using Principal Component Analysis (PCA) with promac oblique rotation in Stata/MP 14.0 (StataCorp, 2015). This was performed on the present analytic cohort, i.e. 402 ÆSOP participants (145 cases, 257 controls) with available neuropsychological data at baseline (all participants), and with entire case history reconstruction at the 10-year follow-up (cases only). Where appropriate, variables were inverse transformed to achieve normality of distribution. The selected cut-off for the variable loadings on the PCA components was 0.30, in line with recommendations that this has practical significance for sample sizes of at least 350 (Hair *et al*., 1998, p. 112). The oblique method was preferred over the varimax solution for its superior capacity to identify a simple factorial structure, particularly in datasets where the latent traits are correlated (Finch, 2006). The Kaiser-Meyer-Olkin (KMO) test of sampling adequacy was used to determine the factorability of the data. To generate composite neuropsychological scores (each reflecting a participant's composite ability across variables with primary loadings on a certain component), we estimated PCA scores using Stata's predict command with the score option immediately after the PCA command. The PCA scores are expressed in standardised units based on linear combinations of the retained components.

#### Comparison of composite neuropsychological scores across study groups

Statistical analysis was performed in Stata/MP 14.0 (StataCorp, 2015). Each of the main and sensitivity (see below) analyses was a multivariable regression analysis with robust standard errors, comparing selected study groups or subgroups in each composite neuropsychological score. All main and sensitivity analyses co-varied for sociodemographic and (where appropriate) clinical variables that were associated with each composite score at *p* *<* 0.1 in preliminary univariable linear regression analyses (see online Supplementary Table S1). Composite neuropsychological scores were compared across the TR, treatment-responder (non-TR) and community control groups, as well as between TR and non-TR cases in the full patient sample and in the diagnostic subgroup with schizophrenia.

Performing separate analyses for cases who were TR from Illness Onset (TRO) and those who had Delayed-Onset Treatment Resistance (DOTR) would enhance the interpretability of our findings. However, this was not possible due to the small size of the DOTR subgroup (*n* = 6). We instead repeated all data analytic steps in sub-analyses which excluded the DOTR patients.

#### Sensitivity analysis

All main statistical analyses uniformly controlled for the effects of ethnicity and education. However, as the TR group had a higher proportion of Black ethnic minorities (47%) than the non-TR (24%) and control (14%) groups (see Results and Table 1), it was important to further address potential confounding influences of language in our analysis. We, therefore, assessed the sensitivity of our findings to excluding all participants who had been born outside the UK, regardless of ethnicity or first language (*n* = 58; 15 non-TR, 6 TR, 37 controls). This left in the analysis an all-UK-born sample who had attended compulsory schooling in the UK.

**Table 1.** Sociodemographic and clinical characteristics in the treatment-responder, treatment-resistant and community control groups

	Treatment-responder (*n* = 113)		Treatment-resistant (*n* = 32)		Community control (*n* = 257)			
	Mean	s.d.	Mean	s.d.	Mean	s.d.	*F*	*p* Value
Education (years)^a^	12.60	2.36	12.10	1.90	13.26	2.55	5.97	**0**.**003**
Age at Assessment (years)^b^	30.75	10.60	26.63	9.07	39.73	13.16	62.81	**<0.0001**
Age at Illness Onset (years)	30.23	10.67	25.41	9.02	–	–	5.71	**0.018**
Days of Untreated Psychosis	197.58	528.52	633.04	1097.00	–	–	4.81	**0.030**
Antipsychotic Defined Daily Dose (DDD)^c^	334.66	238.56	427.81	229.71	–	–	1.89	0.174
Reality Distortion	3.41	2.55	4.00	3.24	–	–	0.70	0.405
Disorganisation	0.69	0.96	0.83	0.82	–	–	0.52	0.471
Negative Symptoms	1.21	1.76	2.38	2.62	–	–	4.36	**0.039**
Mania	1.94	2.73	1.63	2.32	–	–	0.33	0.568
Depression	1.43	1.85	1.09	1.84	–	–	0.70	0.405
	*N*	%	*N*	%	*N*	%	Pearson χ^2^	*p* Value
Gender:^d^							17.39	**<0.0001**
Male	63	55.8	25	78.1	109	42.4		
Female	50	44.2	7	21.9	148	57.6		
Ethnicity: ^e^^,^^f^							Fisher's ^f^	**<0.001**
White	72	63.7	13	40.6	211	82.1		
Black	27	23.9	15	46.9	37	14.4		
Other	14	12.4	4	12.5	9	3.5		
Level of Completed Education:^g^							7.604	0.107
School	65	63.1	19	67.9	131	51.4		
Further	19	18.4	7	25.0	69	27.1		
Higher	19	18.4	2	7.1	55	21.6		
Diagnosis:^f^							Fisher's^f^	**0.004**
Schizophrenia	43	38.1	24	75.0	–	–		
Bipolar Disorder or Mania	24	21.2	2	6.3	–	–		
Depressive Psychosis	18	15.9	2	6.3	–	–		
Other Psychotic Disorder	28	24.8	4	12.5	–	–		
Antipsychotic Type ^c^^,^^f^							Fisher's ^f^	0.725
Typical	22	38.6	8	44.4	–	–		
Atypical	33	57.9	9	50.0	–	–		
Combination of Typical & Atypical	2	3.5	1	5.6	–	–		

Bold denote significance level for *p* values.

a TR, non-TR<CC.

b TR, non-TR<CC, TR<non-TR.

c Data was available for a subset of 75 cases (52%).

d Proportion Male: TR, non-TR>CC; TR>non-TR.

e Proportion Black: TR, non-TR>CC; TR>non-TR.

f As the statistical assumptions for the χ^2^ test were violated, Fisher's Exact Test was performed.

g Level of completed education was missing for 16 participants (4%).

#### Missing data

The level of completeness of sociodemographic and clinical information at the time of the baseline neuropsychological assessment was high (96–100% for socio-demographic variables and 82–100% for clinical variables). The high level of completeness at baseline is partly attributable to the temporal proximity of the sociodemographic, clinical and neuropsychological assessments, which were typically completed within a few days of each other. Antipsychotic medication history was re-constructed in detail at the 10-year follow up. Exact recording of neuropsychological testing dates at baseline was available only in a subset of cases, allowing us to map the participants’ complex medication histories onto the dates of their neuropsychological testing in a subgroup of 75 cases (52%, 57 non-TR, 18 TR). Detailed information on the medication received by this subgroup is presented in online Supplementary Table S2.

## Results

### Representativeness of the analytic cohort

Of the 286 cases who were classified in terms of treatment response at follow-up (see *Methods*), 136 cases (48%) lacked neuropsychological data at baseline, and 5 (2%) had IQ<70, leaving 113 non-TR cases, 32 TR cases (of whom 26: 81% were TRO), and 257 community controls in the present analysis. There were no notable differences in age, gender, DUP or diagnosis between the patient analytic cohort (*n* = 145), and those who lacked neuropsychological data at baseline or met exclusion criteria (*N* = 141). However, the patient analytic cohort (treatment-responders and TR cases combined) comprised a lower proportion of black ethnic minorities (29%) than the cases who lacked neuropsychological data or who met exclusion criteria (51%; χ^2^ = 14.563, *p* = 0.001).

### Sociodemographic and clinical characteristics

The sociodemographic and clinical characteristics of the analytic cohort are presented in Table 1. Compared with controls, both patient groups were younger, had fewer years of education, and a higher proportion of male and Black participants (*p* < 0.01–0.0001). Compared with treatment-responders, TR cases were younger, had a higher proportion of male and Black participants, a higher score on the negative symptom dimension, and a longer duration of untreated psychosis (*p* < 0.05–0.0001) (Table 1). The two patient groups did not differ statistically significantly in illicit substance use [positive lifetime history present in 27 (25%) non-TR- and in 4 (12.5%) TR participants; χ^2^ = 2.237, *p* = 0.135].

### Data reduction and estimation of composite neuropsychological scores

The PCA gave rise to a three-component solution (eigenvalues 1.20–5.78) accounting for 0.65% of the variance. The results of the promax rotation of the solution are presented in Table 2. NART IQ, WAIS-R Vocabulary, WAIS-R Comprehension, Phonological Verbal Fluency and Semantic Verbal Fluency showed primary loadings (0.306–0.520) on Component 1, which was labelled *Verbal Intelligence and Fluency*. WAIS-R Block Design, WAIS-R Digit Symbol and Trail Making (A & B) showed primary loadings (0.332–0.565) on Component 2, which was labelled *Visuospatial Ability and Executive Function*. The immediate and delayed recall trials of the RAVLT showed primary loadings (0.598–0.698) on Component 3, which was labelled *Verbal Memory and Learning*. The Kaiser-Meyer Olkin measure of sampling adequacy indicated that the sample had very high factorability (KMO = 0.875). Composite neuropsychological scores (PCA scores) were generated for the three components and used in the remaining analyses.

**Table 2.** Obliquely rotated component loadings^a^ for 13 neuropsychological variables in the analytic cohort^b^ (*n* = 402)

	Component loadings		
	1	2	3
WAIS-R vocabulary	**0.520**	0.103	0.025
WAIS-R comprehension	0.487	0.124	0.067
NART-R IQ	0.485	0.045	−0.017
Phonological verbal fluency (Letter)	0.322	−0.122	−0.169
Semantic verbal fluency (Category)	0.306	−0.178	−0.119
Trail making A	0.113	**0.565**	0.113
Trail making B	0.064	**0.505**	0.030
WAIS-R digit symbol	0.027	0.416	0.016
WAIS-R block design	0.019	0.332	0.133
RAVLT trials 1–5	0.090	0.046	**0.598**
RAVLT trial 7	−0.047	0.052	**0.698**
WMS-R visual reproduction (Total score)	−0.052	−0.254	0.236
Letter-number span	0.203	−0.126	0.155

NART-R, National Adult Reading Test-Revised; RAVLT, Rey Auditory Verbal Learning Test; WAIS-R, Wechsler Adult Intelligence Scale-Revised.

a Variables with primary loadings of >0.30 are set against a grey background, and high loadings of >0.50 are highlighted in bold font.

b The sample included participants who had undergone neuropsychological testing at baseline, had IQ ⩾70, and could be classified (in the case of patients, retrospectively, i.e. at the 10-year follow-up) as treatment responders (*n* = 113), treatment-resistant (*n* = 32), or community controls (*n* = 257).

### Comparison of composite neuropsychological scores across study groups

**Fig. 1.**
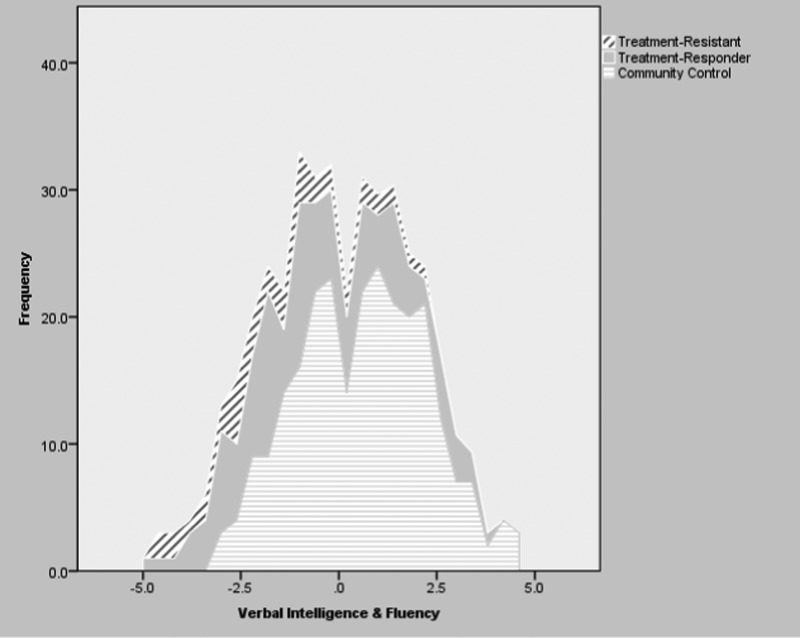
Distribution of Composite Verbal Intelligence & Fluency Scores in the Complete Analytic Cohorts of Treatment-Resistant, Treatment-Responder and Community Control Participants.

Tables 3 and 4 present the means and standard deviations of the composite neuropsychological scores in the three study groups, as well as the results and effect sizes of selected group comparisons. Figure 1 presents the distribution of the composite scores in *Verbal Intelligence and Fluency* in the full analytic cohort divided by study group. Both patient samples were impaired in all composite scores compared with controls (*p⩽*0.001) (Table 3), with moderate to large effect sizes in the treatment-responsive patients and with large to very large effect sizes in the TR cohort.

**Table 3.** Comparison^a^ of composite neuropsychological scores across the treatment-responder, treatment-resistant and community control groups

	Responder (*n* = 113)		Treatment-resistant (*n* = 32)		Community control (*n* = 257)				Regression model^a^			
	Mean	s.d.	Mean	s.d.	Mean	s.d.			*F*_(D.F.)_		*p* Value	
Verbal intelligence & fluency	−0.81	1.81	−1.91	1.68	0.59	1.64			40.15_(7, 386)_			**<0.0001**
Visuospatial ability & executive function	−0.71	1.79	−1.29	2.00	0.47	1.66			37.12_(7, 387)_			**<0.0001**
Verbal memory & learning	−0.37	1.29	−1.13	1.43	0.30	1.37			21.99_(7, 386)_			**<0.0001**
							Post-Hoc comparisons^a^					
	Cohen's *d*^b^		Cohen's *d*^c^				Responder *v.* Control		Treatment-Resistant *v.* Control		Responder *v.* Treatment-Resistant	
	*d*	95% CI	*d*	95% CI			*t*	*p* Value	*t*	*p* Value	*t*	*p* Value
Verbal intelligence & fluency	−0.83	−1.06 to −0.60	−1.52	−1.91 to −1.13			−4.12	**<0.001**	−5.22	**<0.001**	2.35	**0.019**
Visuospatial ability & executive function	−0.69	−0.92 to −0.47	−1.04	−1.41 to −0.66			−6.60	**<0.001**	−5.32	**<0.001**	1.44	0.150
Verbal memory & learning	−0.50	−0.72 to −0.27	−1.04	−1.42 to −0.66			−4.36	**<0.001**	−4.38	**<0.001**	1.94	0.053

Bold denote significance level for *p* values.

a The effect of Group (Treatment Responder, Treatment-Resistant, Community Control) on each Composite Score was examined using multivariable regression analysis with robust standard errors, co-varying for demographic variables that emerged as significant (*p* < 0.05) or suggestive (*p* < 0.1) predictors of each Composite Score in preliminary univariable linear regression analyses (online Supplementary Table S1): Age, Ethnicity, Years of Education (all Composite Scores) and Gender (Verbal Intelligence & Fluency; Verbal Memory & Learning).

b Standardised mean difference the between treatment-responder and community-control groups.

c Standardised mean difference between the treatment-resistant and community-control groups.

**Table 4.** Comparison of composite neuropsychological scores between the treatment-responder and treatment-resistant groups

	Independent effect of group (Responder *v*. Treatment-resistant)					
	Coefficient	Stand. Err.	95% CI		*t*	*p* Value
All diagnoses						
Verbal intelligence & fluency^a^	−0.76	0.32	−1.40	−0.12	−2.35	**0**.**020**
Visuospatial ability & executive function^b^	−0.70	0.57	−1.84	0.45	−1.23	0.226
Verbal memory & learning^c^	−0.40	0.52	−1.46	0.66	−0.76	0.453
Schizophrenia						
Verbal intelligence & fluency^a^	−0.96	0.38	−1.72	−0.20	−2.56	**0.014**
Visuospatial ability & executive function^b^	−1.55	0.61	−1.95	0.28	−2.55	**0.019**
Verbal memory & learning^c^	−0.35	0.74	−1.90	1.20	−0.48	0.640

Bold denote significance level for *p* values.

a The effect of Group (Treatment Responder *v*. Treatment Resistant) on *Verbal Intelligence & Fluency* was examined using multivariable regression analysis with robust standard errors, co-varying for demographic and clinical variables that emerged as significant (*p* < 0.05) or suggestive (*p* < 0.1) predictors of *Verbal Intelligence & Fluency* in preliminary univariable linear regression analyses (online Supplementary Table S1): Age, Gender, Ethnicity, Years of Education, Negative Symptoms, Mania and Depression. The analysis for ‘All Diagnoses’ additionally co-varied for ‘Diagnosis’.

b The effect of Group (Treatment Responder *v.* Treatment Resistant) on *Visuospatial Ability & Executive Function* was examined using multivariable regression analysis with robust standard errors, co-varying for demographic and clinical variables that emerged as significant (*p* < 0.05) or suggestive (*p* < 0.1) predictors of *Visuospatial Ability & Executive Function* in preliminary univariable linear regression analyses (online Supplementary Table S1): Age, Ethnicity, Years of Education, Age at Illness Onset, Negative Symptoms, Mania, Medication Dose (expressed in Defined Daily Dose units) and Illicit Substance Use [positive/negative lifetime history of, based on information collected from relatives or carers, the Schedules for Clinical Assessment in Neuropsychiatry (SCAN; WHO, 1992*b*), clinical case notes and an extended version of the WHO Life Chart Schedule (WHO, 1992*c*)]. The analysis for ‘All Diagnoses’ additionally co-varied for ‘Diagnosis’.

c The effect of Group (Treatment Responder *v.* Treatment Resistant) on *Verbal Memory & Learning* was examined using multivariable regression analysis with robust standard errors, co-varying for demographic and clinical variables that emerged as significant (*p* < 0.05) or suggestive (*p* < 0.1) predictors of *Verbal Memory & Learning* in preliminary univariable linear regression analyses (online Supplementary Table S1): Age, Gender, Ethnicity, Years of Education, Duration of Untreated Psychosis, Reality Distortion, Negative Symptoms, Mania and Medication Dose (expressed in Defined Daily Dose units). The analysis for ‘All Diagnoses’ additionally co-varied for ‘Diagnosis’.

Compared to non-TR patients, the TR cases performed worse in *Verbal Intelligence and Fluency* in all main analyses (*p* < 0.05) (Tables 3 and 4; Figure 1), sub-analyses (*p* < 0.01–0.001) (excluding DOTR case: online Supplementary Tables S3 and S4), and sensitivity analyses (*p* < 0.05) (excluding non-UK-born participants: online Supplementary Tables S5 and S6).

In the schizophrenia subgroup, TR cases performed worse than non-TR patients in *Visuospatial Ability and Executive* Function (*p* < 0.05) (Table 4), but the finding did not persist in our sub-analyses or sensitivity analyses (online Supplementary Tables S3–S6).

## Discussion

### Summary of findings

Our analysis of baseline data from the ÆSOP-10 longitudinal, population-based study of FEP provides a snapshot of the neuropsychological function at the earliest stages of TR psychosis. Both TR and treatment-responsive patients with schizophrenia and other psychoses showed generalised neuropsychological deficits in verbal intelligence and fluency, visuospatial abilities and executive function, and verbal memory and learning compared with community controls. Furthermore, TR patients showed, on average, impairments in verbal intelligence and fluency compared with treatment responders, replicating previous findings (Joober *et al*., 2002; Frydecka *et al*., 2016). This differential was an enduring finding of our analyses – evident for individuals with schizophrenia, those with any psychoses, psychotic and schizophrenia patients born in the UK, and after excluding cases with delayed-onset treatment resistance.

### Methodological considerations

This is the first longitudinal, population-based study of FEP that compared baseline neuropsychological function across patients with longitudinally-defined TR- and non-TR psychosis and community controls. The study draws on unique methodological advantages: The epidemiological source and robust size of the combined patient and community control groups increased the representativeness of our cohort and generalisability of our findings, whilst facilitating stringent statistical controls (including a sensitivity analysis) for a wide array of clinical, sociodemographic, and language confounders. Analysing neuropsychological data from the first episode minimised information bias (i.e. examiners did not know the participants’ treatment response status at the time of testing) and reduced differentiation in clinical features between the two patient groups. Specifically, in contrast to all previous neuropsychological studies of TRS (Joober *et al*., 2002; Bourque *et al*., 2013; de Bartolomeis *et al*., 2013; Anderson *et al*., 2015; Frydecka *et al*., 2016; Iasevoli *et al*., 2016), our patient groups did not seem to differ notably in treatment profiles or medication doses at the time of testing (based on a subgroup analysis). These aspects served to further reduce confounding in our analysis.

Methodological limitations of our study include loss to follow up; limitations on clinical data accuracy associated with case history reconstruction; the availability of baseline neuropsychological testing dates for only a subgroup of patients; the lack of screening for family history of psychosis in community controls; and the moderate size of the TR cohort. A deficit in verbal intelligence and fluency in TR patients compared with responders was a highly consistent finding across our main analyses, sub-analyses and sensitivity analyses. However, we cannot exclude the possibility that additional deficits are integral to TR psychosis, particularly in relation to schizophrenia (discussed below). Our selected cut-off for factor loadings (0.30) is among the lowest reported in the literature (Peres-Neto *et al*., 2003), and may have reduced the clarity of the PCA components. Using a 0.40 cut-off would not have changed the pattern of findings (data available upon request). Finally, our analyses included baseline diagnoses. As with the extended ÆSOP sample (Heslin *et al*., 2015), most schizophrenia patients (77%) and 40% of those with ‘other’ diagnoses in our analytic cohort were classified as having schizophrenia at 10 years. Using diagnostic classifications at follow up, 31.5% of schizophrenia patients would have been classified as TR compared with 35.8% using baseline diagnoses.

### TR psychosis as a severe neurodevelopmental variant of psychosis

The neurodevelopmental theory of schizophrenia posits the existence of a neurodevelopmental subtype of schizophrenia, which is the end product of aberrant neurodevelopmental processes unfolding from conception or early life (Murray *et al*., 1992). It has been suggested that primary TRS has a distinct neurodevelopmental origin, while secondary TRS may arise through the induction of dopamine super-sensitivity, or after periods of relapse, although a later emergence of an intrinsic treatment resistance, or a combination of underlying factors cannot be ruled out (Lally *et al*., 2016; Demjaha *et al*., 2017; Gillespie *et al*., 2017). The clinical and demographic profiles of the TR cases in the present study largely encapsulate the defining features of ‘neurodevelopmental schizophrenia’ – younger, ‘more male’, with an earlier age of onset, more severe negative symptoms, more severe cognitive impairment and a longer duration of untreated psychosis (Murray *et al*., 1992). Three further observations suggest a pathogenic origin for the observed verbal deficit in the TR group compared with treatment-responsive individuals. Firstly, the impairment was established by the first episode, arguing against the deficit being caused by non-remitting psychosis. Secondly, Vocabulary and NART-R, two tasks with primary loadings on Verbal Intelligence and Fluency, are reliable tests of premorbid ability, and are both resistant to brain pathological changes (Bright *et al*., 2002; de Oliveira *et al*., 2014). Finally, the pattern of deficits in verbal intelligence and fluency was accentuated (deficits were significant at a lower level of statistical significance) after removing cases with secondary treatment resistance.

Black participants were over-represented among TR patients. Although Black ethnicity is not a defining feature of ‘neurodevelopmental schizophrenia’ (Murray *et al*., 1992), the finding is in keeping with evidence that treatment resistance is associated with early first contact with psychiatric services (<20 years), and more so in Black (OR 3.71) than in White (OR 1.60) patients (Lally *et al*., 2016). Indeed, a closer look at our data revealed that only 9.4% of White patients, but 21.4% of Black patients had the first contact with psychiatric services before age 20, which may have increased disproportionately the outcome of treatment resistance in the Black ethnic group.

### TR schizophrenia

An additional deficit in visuospatial ability and executive function emerged in TR- compared with non-TR patients in the schizophrenia subgroup. This finding did not generalise to the ‘all-diagnoses’ group and did not persist in our sub-analyses or sensitivity analyses. The finding is consistent with evidence that deficits in executive function, processing speed and verbal memory, albeit less salient in other diagnostic categories of psychosis, are prototypical of schizophrenia (Kravariti *et al*., 2009*a*, 2009*b*; Zanelli *et al*., 2010). As the size of the schizophrenia subgroup in the present study was modest, it is important to explore the significance of executive function and verbal memory deficits in TR schizophrenia in larger studies.

### Integrating neurodevelopmental and glutamatergic hypotheses of TR psychosis

Some recent findings implicate glutamate rather than dopamine as the primary neurotransmitter system impaired in TR schizophrenia (Demjaha *et al*., 2012, 2014; Gillespie *et al*., 2017). Glutamate plays an important role in several language-related neurodevelopmental processes. This highlights the possibility that core deficits in verbal intelligence and fluency, a neurodevelopmental aetiology, and a primary glutamatergic dysfunction may converge in a single model of TR psychosis. Several lines of evidence support this possibility: pre-reading language abilities (e.g. phonological processing) show significant correlations with glutamate in the anterior cingulate of healthy preschool-aged children (Lebel *et al*., 2016); microdeletions in glutamate receptors have been implicated in developmental delays predominantly affecting language and fine motor skills (Takenouchi *et al*., 2014); the high-risk metabotropic glutamate receptor 3 (GRM3) haplotype is associated with schizophrenia, as well as with deficits in verbal fluency and verbal list-learning (Spangaro *et al*., 2012); and poor-functioning subjects at ultra-high-risk for psychosis show a negative relationship between thalamic glutamate levels and prefrontal-striatal activation during a verbal fluency task (Allen *et al*., 2015).

### Clinical and research implications

Neuropsychological deficits weigh disproportionally on the psychosocial and functional toll of psychosis (Kaneda *et al*., 2010; Shamsi *et al*., 2011; Iasevoli *et al*., 2016). Encouragingly, verbal fluency and executive function deficits, which differentiated TR from non-TR patients in the present study, do not seem refractory to pharmacological interventions. Indeed, there is strong evidence that clozapine improves attention and verbal fluency, and moderate evidence that it improves some types of executive function (Meltzer and McGurk, 1999; Woodward *et al*., 2005). In the only studies to report equivalent verbal performances in TRS- and non-TRS patients to date, TR cases were uniformly treated with clozapine (Bourque *et al*., 2013; Anderson *et al*., 2015). These findings re-iterate the necessity of timely detection and tailored pharmacological interventions as early as possible in the course of TR psychosis (Lally and MacCabe, 2016). They further highlight the importance of neuropsychological constructs in designing multimodal research and clinical approaches to improving prognosis and personalised treatment (Gilbert *et al*., 2014).

## Conclusion

A constitutional deficit in verbal intelligence and fluency, significantly exceeding – at a group level – the levels manifest in the general population of patients with psychoses, is a phenotypic indicator of TR psychosis. Our findings are in keeping with emerging evidence that TR psychosis is a pathogenically distinct and severe variant, embedded in aberrant neurodevelopmental processes.

## Supplementary material

For supplementary material accompanying this paper visit https://doi.org/10.1017/S0033291718002957. click here to view supplementary material
