# Supplementary material for: Neuropsychological function at first episode in treatment-resistant psychosis: findings from the ÆSOP-10 study
Source: Psychol Med. 2018 Oct 23;49(12):2100–10. doi: 10.1017/S0033291718002957 (PMC6712950; doi:10.1017/S0033291718002957)
Supplement: Supplementary file 1 [file S0033291718002957sup001.docx]

**Table S1** Predictive Effects of Sociodemographic and Clinical Variables on Neuropsychological Composite Scores Examined in Preliminary Univariable Linear Regression Models ^a^

|  |  | **Verbal Intelligence & Fluency** | |  | **Visuospatial Ability & Executive Function** | |  | **Verbal Memory & Learning** | |
| --- | --- | --- | --- | --- | --- | --- | --- | --- | --- |
|  |  | **F_(D.F.)_** | ***P≤*** |  | **F_(D.F.)_** | ***P≤*** |  | **F_(D.F.)_** | ***P≤*** |
|  |  |  |  |  |  |  |  |  |  |
| ***All Participants (n=402)*** |  |  |  |  |  |  |  |  |  |
|  |  |  |  |  |  |  |  |  |  |
| **Years of Age at Assessment ^b^** |  | 34.30_(1, 400)_ | 0.0001 |  | 7.98_(1, 400)_ | 0.005 |  | 4.56_(1, 400)_ | 0.033 |
| **Gender** |  | 4.55_(1, 400)_ | 0.034 |  | 1.83_(1, 400)_ | 0.177 |  | 14.47_(1, 400)_ | 0.001 |
| **Ethnicity** |  | 34.55_(2, 399)_ | 0.0001 |  | 15.26_(2, 399)_ | 0.0001 |  | 11.93_(2, 399)_ | 0.0001 |
| **Years of Education** |  | 93.99_(1, 392)_ | 0.0001 |  | 58.65_(1, 392)_ | 0.0001 |  | 58.39_(1, 392)_ | 0.0001 |
|  |  |  |  |  |  |  |  |  |  |
| ***Patients (n=145)*** |  |  |  |  |  |  |  |  |  |
|  |  |  |  |  |  |  |  |  |  |
| **Diagnosis** |  | 3.81_(3, 141)_ | 0.012 |  | 4.01_(3, 141)_ | 0.009 |  | 3.82_(3, 141)_ | 0.011 |
| **Years of Age at Illness Onset ^b^** |  | 0.40_(1, 127)_ | 0.528 |  | 6.66_(1, 127)_ | 0.011 |  | 0.02_(1, 127)_ | 0.877 |
| **Duration of Untreated Psychosis** |  | 0.52_(1, 140)_ | 0.471 |  | 0.13_(1, 140)_ | 0.720 |  | 11.78_(1, 140)_ | 0.001 |
| **Reality Distortion** |  | 1.52_(1, 117)_ | 0.220 |  | 1.46_(1, 117)_ | 0.230 |  | 3.27_(1, 117)_ | 0.073 |
| **Disorganisation** |  | 1.40_(1, 117)_ | 0.239 |  | 0.03_(1, 117)_ | 0.862 |  | 2.16_(1, 117)_ | 0.144 |
| **Negative Symptoms** |  | 3.69_(1, 117)_ | 0.057 |  | 6.39_(1, 117)_ | 0.013 |  | 10.17_(1, 117)_ | 0.002 |
| **Mania** |  | 5.44_(1, 117)_ | 0.021 |  | 4.39_(1, 117)_ | 0.038 |  | 4.01_(1, 117)_ | 0.048 |
| **Depression** |  | 3.61_(1, 117)_ | 0.060 |  | 0.45_(1, 117)_ | 0.502 |  | 1.56_(1, 117)_ | 0.214 |
| **Antipsychotic Type ^c^** |  | 1.62_(1, 73)_ | 0.207 |  | 1.36_(1, 73)_ | 0.248 |  | 0.02_(1, 73)_ | 0.876 |
| **Antipsychotic Defined Daily Dose ^d^** |  | 2.77_(1, 66)_ | 0.101 |  | 5.43_(1, 66)_ | 0.023 |  | 4.77_(1, 66)_ | 0.033 |
| **Illicit Substance Use ^e^** |  | 1.42_(1, 138)_ | 0.236 |  | 3.21_(1, 138)_ | 0.076 |  | 0.15_(1, 138)_ | 0.703 |

The *P*-values of statistically significant (*P*<0.05) and suggestive (*P*<0.1) results are set against dark-grey and light-grey backgrounds, respectively.
^a^ The results of the univariable linear regression models informed the selection of covariates for the multivariable regression models of the main statistical analysis. Statistically significant or suggestive predictors were included as covariates in multivariable regression models examining differences in neuropsychological composite scores across the treatment responder, treatment resistant and community control groups, or between the treatment responder and treatment resistant groups in the main statistical analysis. ^b^ Due to the multi-collinearity of age and age of illness onset (Variance Inflation Factors: 14.79 and 13.32, respectively), only age (not age of onset), was included as covariate in the multivariable regression model for Visuospatial Ability & Executive Function. ^c^ First-generation versus second-generation antipsychotic. ^d^ Dose was expressed in Defined Daily Dose (DDD) units (Patel *et al.* 2013). ^e^ Positive/negative lifetime history of, based on information collected from relatives or carers, the Schedules for Clinical Assessment in Neuropsychiatry (SCAN; WHO, 1992a), clinical case notes and an extended version of the WHO Life Chart Schedule (WHO, 1992b).

**Table S2** Daily Antipsychotic Medication Regimes in a Subset of Participants with First Episode Psychosis (n=75)

|  | **Antipsychotic Medication 1** | |  | **Antipsychotic Medication 2** | |
| --- | --- | --- | --- | --- | --- |
| **SUBJECT ID** | **Name** | **Defined Daily Dose ^a^** |  | **Name** | **Defined Daily Dose ^a^** |
|  | | | | | |
| ***TREATMENT-RESPONDER*** | | | | | |
| 1 | Droperidol |  |  |  |  |
| 2 | Haloperidol | 187.50 |  |  |  |
| 3 | Trifluoperazine | 150.00 |  |  |  |
| 4 | Sertindole | 300.00 |  |  |  |
| 5 | Risperidone | 480.00 |  |  |  |
| 6 | Olanzapine | 300.00 |  |  |  |
| 7 | Chlorpromazine | 200.00 |  |  |  |
| 8 | Olanzapine | 300.00 |  |  |  |
| 9 | Thioridazine | 75.00 |  |  |  |
| 10 | Trifluoperazine | 75.00 |  |  |  |
| 11 | Olanzapine | 300.00 |  |  |  |
| 12 | Olanzapine | 300.00 |  |  |  |
| 13 | Olanzapine | 450.00 |  |  |  |
| 14 | Olanzapine | 150.00 |  |  |  |
| 15 | Olanzapine | 600.00 |  |  |  |
| 16 | Olanzapine | 300.00 |  |  |  |
| 17 | Haloperidol | 1125.00 |  | Olanzapine | 300.00 |
| 18 | Risperidone | 300.00 |  |  |  |
| 19 | Olanzapine | 300.00 |  | Flupenthixol |  |
| 20 | Risperidone | 480.00 |  | Olanzapine | 600.00 |
| 21 | Sulpiride | 300.00 |  |  |  |
| 22 | Risperidone | 480.00 |  |  |  |
| 23 | Droperidol |  |  |  |  |
| 24 | Amisulpride | 150.00 |  |  |  |
| 25 | Sulpiride | 300.00 |  |  |  |
| 26 | Risperidone | 120.00 |  |  |  |
| 27 | Olanzapine | 600.00 |  | Quetiapine | 300.00 |
| 28 | Risperidone | 240.00 |  |  |  |
| 29 | Olanzapine | 600.00 |  |  |  |
| 30 | Olanzapine | 450.00 |  |  |  |
| 31 | Olanzapine | 300.00 |  |  |  |
| 32 | Risperidone | 180.00 |  |  |  |
| 33 | Olanzapine | 300.00 |  |  |  |
| 34 | Olanzapine | 300.00 |  |  |  |
| 35 | Trifluoperazine | 150.00 |  |  |  |
| 36 | Olanzapine | 300.00 |  | Risperidone | 180.00 |
| 37 | Chlorpromazine | 400.00 |  |  |  |
| 38 | Olanzapine | 600.00 |  |  |  |
| 39 | Olanzapine | 300.00 |  |  |  |
| 40 | Olanzapine | 450.00 |  |  |  |
| 41 | Olanzapine | 450.00 |  |  |  |
| 42 | Quetiapine | 375.00 |  |  |  |
| 43 | Olanzapine | 300.00 |  |  |  |
| 44 | Quetiapine | 300.00 |  |  |  |
| 45 | Risperidone | 180.00 |  |  |  |
| 46 | Olanzapine | 300.00 |  |  |  |
| 47 | Thioridazine | 20.00 |  |  |  |
| 48 | Haloperidol | 375.00 |  |  |  |
| 49 | Trifluoperazine | 75.00 |  |  |  |
| 50 | Trifluoperazine | 60.00 |  | Thioridazine | 100.00 |
| 51 | Flupenthixol |  |  |  |  |
| 52 | Chlorpromazine | 300.00 |  |  |  |
| 53 | Trifluoperazine | 225.00 |  |  |  |
| 54 | Chlorpromazine | 150.00 |  |  |  |
| 55 | Thioridazine | 75.00 |  |  |  |
| 56 | Haloperidol |  |  |  |  |
| 57 | Haloperidol | 562.50 |  |  |  |
|  | | | | | |
| ***TREATMENT-RESISTANT*** | | | | | |
| 58 | Sulpiride | 150.00 |  |  |  |
| 59 | Flupenthixol |  |  |  |  |
| 60 | Olanzapine | 450.00 |  |  |  |
| 61 | Olanzapine | 600.00 |  |  |  |
| 62 | Sulpiride | 600.00 |  |  |  |
| 63 | Olanzapine | 300.00 |  |  |  |
| 64 | Olanzapine | 300.00 |  |  |  |
| 65 | Olanzapine |  |  |  |  |
| 66 | Quetiapine | 450.00 |  | Haloperidol | 375.00 |
| 67 | Trifluoperazine | 225.00 |  |  |  |
| 68 | Risperidone | 480.00 |  |  |  |
| 69 | Olanzapine | 450.00 |  |  |  |
| 70 | Flupenthixol |  |  |  |  |
| 71 | Olanzapine | 300.00 |  |  |  |
| 72 | Risperidone | 240.00 |  |  |  |
| 73 | Trifluoperazine | 337.50 |  |  |  |
| 74 | Zuclopenthixol | 200.00 |  |  |  |
| 75 | Trifluoperazine | 300.00 |  |  |  |

^a^ Patel *et al*. (2013)

**Table S3** Comparison ^a^ of Composite Neuropsychological Scores across the Treatment-Responder, Treatment-Resistant and Community Control Groups after excluding participants with Delayed-Onset Treatment Resistance (n=6)

|  | **Treatment-Responder** | |  | **Treatment-Resistant** | |  | **Community Control** | |  | |  | |  | |
| --- | --- | --- | --- | --- | --- | --- | --- | --- | --- | --- | --- | --- | --- | --- |
|  | (n=113) | |  | (n=26) | |  | (n=257) | |  | |  | |  | |
|  |  |  |  |  |  |  |  |  | **Regression Model ^a^** | | | | | |
|  | **Mean** | **SD** |  | **Mean** | **SD** |  | **Mean** | **SD** |  | | **F_(D.F.)_** | | ***P≤*** | |
| **Verbal Intelligence & Fluency** | -0.81 | 1.81 |  | -2.01 | 1.82 |  | 0.59 | 1.64 |  | | 40.62_(7, 380)_ | | **0.0001** | |
| **Visuospatial Ability & Executive Function** | -0.71 | 1.79 |  | -1.32 | 1.99 |  | 0.47 | 1.66 |  | | 34.51_(6, 381)_ | | **0.0001** | |
| **Verbal Memory & Learning** | -0.37 | 1.29 |  | -1.08 | 1.44 |  | 0.30 | 1.37 |  | | 20.95_(7, 380)_ | | **0.0001** | |
|  |  |  |  |  |  |  |  |  |  | |  | |  | |
|  |  |  |  |  |  |  |  |  | **Post-Hoc Comparisons ^a^** | | | | | |
|  | **Cohen’s *d* ^b^** | |  | **Cohen’s *d* ^c^** | |  |  |  | **Responder vs. Control** | | **Treatment Resistant vs. Control** | | **Responder vs. Treatment Resistant** | |
|  | ***d*** | **95% CI** | | ***d*** | **95% CI** | |  |  | **t** | ***P≤*** | **t** | ***P≤*** | **t** | ***P≤*** |
| **Verbal Intelligence & Fluency** | -0.83 | -1.06 -0.60 | | -1.57 | -1.99 -1.14 | |  |  | -4.06 | **0.001** | -4.93 | **0.001** | 2.45 | **0.001** |
| **Visuospatial Ability & Executive Function** | -0.69 | -0.92 -0.47 | | -1.06 | -1.47 -0.64 | |  |  | -6.59 | **0.001** | -4.96 | **0.001** | 1.49 | 0.138 |
| **Verbal Memory & Learning** | -0.50 | -0.72 -0.27 | | -1.00 | -1.41 -0.59 | |  |  | -4.27 | **0.001** | -3.79 | **0.001** | 1.60 | 0.111 |
|  |  |  |  |  |  |  |  |  |  |  |  |  |  |  |

^a^ The effect of Group (Treatment Responder, Treatment Resistant, Community Control) on each Composite Score was examined using multivariable regression analysis with robust standard errors, co-varying for demographic variables that emerged as significant (P<0.05) or suggestive (P<0.1) predictors of each Composite Score in preliminary univariable linear regression analyses (Supplementary Table S1): Age, Ethnicity, Years of Education (all Composite Scores) and Gender (Verbal Intelligence & Fluency; Verbal Memory & Learning). ^b^ Standardised mean difference the between treatment-responder and community-control groups; ^c^ Standardised mean difference between the treatment-resistant and community-control groups.

**Table S4** Comparison of Composite Neuropsychological Scores between the Treatment-Responder and Treatment-Resistant Groups after excluding participants with Delayed-Onset Treatment Resistance (n=6)

|  |  | **Independent Effect of Group (Treatment-Responder vs. Treatment-Resistant)** | | | | | | | | |  |
| --- | --- | --- | --- | --- | --- | --- | --- | --- | --- | --- | --- |
|  |  | **Coefficient** |  | **Stand. Err.** |  | **95% CI** | |  | **t** | ***P≤*** |  |
|  |  |  | | | | | | | | |  |
|  |  | ***All Psychoses*** | | | | | | | | |  |
|  |  |  | | | | | | | | |  |
| **Verbal Intelligence & Fluency *^a^*** |  | -0.97 |  | 0.35 |  | -1.67 | -0.27 |  | -2.74 | **0.007** |  |
| **Visuospatial Ability & Executive Function *^b^*** |  | -0.57 |  | 0.67 |  | -1.92 | 0.78 |  | -0.85 | 0.400 |  |
| **Verbal Memory *^c^*** |  | -0.13 |  | 0.51 |  | -1.18 | 0.91 |  | -0.26 | 0.795 |  |
|  |  |  |  |  |  |  |  |  |  |  |  |
|  | ***Schizophrenia*** | | | | | | | | | |  |
|  |  | | | | | | | | | |  |
| **Verbal Intelligence & Fluency *^a^*** |  | -1.47 |  | 0.37 |  | -2.21 | -0.72 |  | -3.99 | **0.001** |  |
| **Visuospatial Ability & Executive Function *^b^*** |  | -1.22 |  | 0.82 |  | -2.95 | 0.52 |  | -1.49 | 0.157 |  |
| **Verbal Memory *^c^*** |  | 0.09 |  | 1.05 |  | -2.16 | 2.33 |  | 0.08 | 0.936 |  |
|  |  |  |  |  |  |  |  |  |  |  |  |

^a^ The effect of Group (Treatment Responder vs. Treatment Resistant) on *Verbal Intelligence & Fluency* was examined using multivariable regression analysis with robust standard errors, co-varying for demographic and clinical variables that emerged as significant (*P*<0.05) or suggestive (*P*<0.1) predictors of *Verbal Intelligence & Fluency* in preliminary univariable linear regression analyses (**Supplementary Table S1**): Age, Gender, Ethnicity, Years of Education, Negative Symptoms, Mania and Depression. The analysis for ‘All Diagnoses’ additionally co-varied for ‘Diagnosis’.

^b^ The effect of Group (Treatment Responder vs. Treatment Resistant) on *Visuospatial Ability & Executive Function* was examined using multivariable regression analysis with robust standard errors, co-varying for demographic and clinical variables that emerged as significant (*P*<0.05) or suggestive (*P*<0.1) predictors of *Visuospatial Ability & Executive Function* in preliminary univariable linear regression analyses (**Supplementary Table S1**): Age, Ethnicity, Years of Education, Age at Illness Onset, Negative Symptoms, Mania, Medication Dose (expressed in Defined Daily Dose units) and Illicit Substance Use [positive/negative lifetime history of, based on information collected from relatives or carers, the Schedules for Clinical Assessment in Neuropsychiatry (SCAN; WHO, 1992a), clinical case notes and an extended version of the WHO Life Chart Schedule (WHO, 1992b)]. The analysis for ‘All Diagnoses’ additionally co-varied for ‘Diagnosis’.

^c^ The effect of Group (Treatment Responder vs. Treatment Resistant) on *Verbal Memory & Learning* was examined using multivariable regression analysis with robust standard errors, co-varying for demographic and clinical variables that emerged as significant (*P*<0.05) or suggestive (*P*<0.1) predictors of *Verbal Memory & Learning* in preliminary univariable linear regression analyses (**Supplementary Table S1)**: Age, Gender, Ethnicity, Years of Education, Duration of Untreated Psychosis, Reality Distortion, Negative Symptoms, Mania and Medication Dose (expressed in Defined Daily Dose units). The analysis for ‘All Diagnoses’ additionally co-varied for ‘Diagnosis’.

**Table S5** Comparison ^a^ of Composite Neuropsychological Scores across the Treatment-Responder, Treatment-Resistant and Community Control Groups after excluding participants born outside the UK (n=58)

|  | **Responder** | |  | **Treatment-Resistant** | |  | **Community Control** | |  | |  | |  | |
| --- | --- | --- | --- | --- | --- | --- | --- | --- | --- | --- | --- | --- | --- | --- |
|  | (n=89) | |  | (n=22) | |  | (n=220) | |  | |  | |  | |
|  |  |  |  |  |  |  |  |  | **Regression Model ^a^** | | | | | |
|  | **Mean** | **SD** |  | **Mean** | **SD** |  | **Mean** | **SD** |  | | **F_(D.F.)_** | | ***P≤*** | |
| **Verbal Intelligence & Fluency** | -0.56 | 1.83 |  | -1.86 | 1.64 |  | 0.66 | 1.59 |  | | 34.19_(7, 328)_ | | **0.0001** | |
| **Visuospatial Ability & Executive Function** | -0.54 | 1.79 |  | -1.09 | 2.12 |  | 0.60 | 1.62 |  | | 25.10_(6, 329)_ | | **0.0001** | |
| **Verbal Memory & Learning** | -0.33 | 1.38 |  | -1.09 | 1.52 |  | 0.34 | 1.31 |  | | 16.66_(7, 328)_ | | **0.0001** | |
|  |  |  |  |  |  |  |  |  |  | |  | |  | |
|  |  |  |  |  |  |  |  |  | **Post-Hoc Comparisons ^a^** | | | | | |
|  | **Cohen’s *d* ^b^** | |  | **Cohen’s *d* ^c^** | |  |  |  | **Responder vs. Control** | | **Treatment Resistant vs. Control** | | **Responder vs. Treatment Resistant** | |
|  | ***d*** | **95% CI** | | ***d*** | **95% CI** | |  |  | **t** | ***P≤*** | **t** | ***P≤*** | **t** | ***P≤*** |
| **Verbal Intelligence & Fluency** | -0.73 | -0.99 -0.48 | | -1.58 | -2.04 -1.12 | |  |  | -3.61 | **0.001** | -4.69 | **0.001** | 2.32 | **0.021** |
| **Visuospatial Ability & Executive Function** | -0.68 | -0.93 -0.43 | | -1.01 | -1.46 -0.56 | |  |  | -6.41 | **0.001** | -4.55 | **0.001** | 1.19 | 0.234 |
| **Verbal Memory & Learning** | -0.50 | -0.75 -0.25 | | -1.08 | -1.52 -0.63 | |  |  | -3.80 | **0.001** | -4.00 | **0.001** | 1.84 | 0.066 |
|  |  |  |  |  |  |  |  |  |  |  |  |  |  |  |

^a^ The effect of Group (Treatment Responder, Treatment Resistant, Community Control) on each Composite Score was examined using multivariable regression analysis with robust standard errors, co-varying for demographic variables that emerged as significant (P<0.05) or suggestive (P<0.1) predictors of each Composite Score in preliminary univariable linear regression analyses (Supplementary Table S1): Age, Ethnicity, Years of Education (all Composite Scores) and Gender (Verbal Intelligence & Fluency; Verbal Memory & Learning). ^b^ Standardised mean difference the between treatment-responder and community-control groups; ^c^ Standardised mean difference between the treatment-resistant and community-control groups.

**Table S6** Comparison of Composite Neuropsychological Scores between the Treatment-Responder and Treatment-Resistant Groups after excluding participants born outside the UK (n=58)

|  |  | **Independent Effect of Group (Responder vs. Treatment Resistant)** | | | | | | | | |  |
| --- | --- | --- | --- | --- | --- | --- | --- | --- | --- | --- | --- |
|  |  | **Coefficient** |  | **Stand. Err.** |  | **95% CI** | |  | **t** | ***P≤*** |  |
|  |  |  | | | | | | | | |  |
|  |  | ***All Psychoses*** | | | | | | | | |  |
|  |  |  | | | | | | | | |  |
| **Verbal Intelligence & Fluency *^a^*** |  | -0.97 |  | 0.38 |  | -1.73 | -0.22 |  | -2.58 | **0.012** |  |
| **Visuospatial Ability & Executive Function *^b^*** |  | -0.57 |  | 0.65 |  | -1.90 | 0.76 |  | -0.87 | 0.389 |  |
| **Verbal Memory *^c^*** |  | -0.05 |  | 0.57 |  | -1.23 | 1.12 |  | -0.09 | 0.927 |  |
|  |  |  |  |  |  |  |  |  |  |  |  |
|  | ***Schizophrenia*** | | | | | | | | | |  |
|  |  | | | | | | | | | |  |
| **Verbal Intelligence & Fluency *^a^*** |  | -1.04 |  | 0.48 |  | -2.02 | -0.05 |  | -2.15 | **0.039** |  |
| **Visuospatial Ability & Executive Function *^b^*** |  | -1.01 |  | 0.96 |  | -3.10 | 1.08 |  | -1.06 | 0.311 |  |
| **Verbal Memory *^c^*** |  | -0.01 |  | 0.98 |  | -2.16 | 2.15 |  | -0.01 | 0.996 |  |
|  |  |  |  |  |  |  |  |  |  |  |  |

^a^ The effect of Group (Treatment Responder vs. Treatment Resistant) on *Verbal Intelligence & Fluency* was examined using multivariable regression analysis with robust standard errors, co-varying for demographic and clinical variables that emerged as significant (*P*<0.05) or suggestive (*P*<0.1) predictors of *Verbal Intelligence & Fluency* in preliminary univariable linear regression analyses (**Supplementary Table S1**): Age, Gender, Ethnicity, Years of Education, Negative Symptoms, Mania and Depression. The analysis for ‘All Diagnoses’ additionally co-varied for ‘Diagnosis’.

^b^ The effect of Group (Treatment Responder vs. Treatment Resistant) on *Visuospatial Ability & Executive Function* was examined using multivariable regression analysis with robust standard errors, co-varying for demographic and clinical variables that emerged as significant (*P*<0.05) or suggestive (*P*<0.1) predictors of *Visuospatial Ability & Executive Function* in preliminary univariable linear regression analyses (**Supplementary Table S1**): Age, Ethnicity, Years of Education, Age at Illness Onset, Negative Symptoms, Mania, Medication Dose (expressed in Defined Daily Dose units) and Illicit Substance Use [positive/negative lifetime history of, based on information collected from relatives or carers, the Schedules for Clinical Assessment in Neuropsychiatry (SCAN; WHO, 1992a), clinical case notes and an extended version of the WHO Life Chart Schedule (WHO, 1992b)]. The analysis for ‘All Diagnoses’ additionally co-varied for ‘Diagnosis’.

^c^ The effect of Group (Treatment Responder vs. Treatment Resistant) on *Verbal Memory & Learning* was examined using multivariable regression analysis with robust standard errors, co-varying for demographic and clinical variables that emerged as significant (*P*<0.05) or suggestive (*P*<0.1) predictors of *Verbal Memory & Learning* in preliminary univariable linear regression analyses (**Supplementary Table S1)**: Age, Gender, Ethnicity, Years of Education, Duration of Untreated Psychosis, Reality Distortion, Negative Symptoms, Mania and Medication Dose (expressed in Defined Daily Dose units). The analysis for ‘All Diagnoses’ additionally co-varied for ‘Diagnosis’.

**REFERENCES**

**Patel MX, Arista I, Taylor M, Barnes TR** (2013). How to compare doses of different antipsychotics: a systematic review of methods. *Schizophrenia Research* **149**, 141-148.

**World Health Organization (WHO)** (1992a). *SCAN (Schedules for Clinical Assessment in Neuropsychiatry)*. World Health Organization: Geneva.

**World Health Organization (WHO)** (1992b). The Life Chart Schedule. Developed by Ezra Susser, Sarah Conover, Carole Siegel and an International Team of WHO Investigators. World Health Organization: Geneva.
